# A novel small-molecule inhibitor GSK-F1 confers radiosensitivity by inhibiting the NSUN2/TP53/RAD51 axis-mediated DNA homologous recombination repair in nasopharyngeal carcinoma

**DOI:** 10.7150/ijbs.130087

**Published:** 2026-03-30

**Authors:** Lemei Zheng, Mengna Li, Xiaolong Li, Jianxia Wei, Changning Xue, Qingqing Wei, Yumei Duan, Huizhen Xin, Zubing Wu, Ting Zeng, Wei Xiong, Songqing Fan, Ming Zhou, Hongyu Deng

**Affiliations:** 1 NHC Key Laboratory of Carcinogenesis, Hunan Key Laboratory of Oncotarget Gene, Hunan Cancer Hospital and the Affiliated Cancer Hospital of Xiangya School of Medicine, Central South University, Changsha 410013, China.; 2 Cancer Research Institute, School of Basic Medical Sciences, Central South University, Changsha 410078, China.; 3 The Key Laboratory of Carcinogenesis and Cancer Invasion of the Chinese Ministry of Education, Central South University, Changsha 410078, China.; 4 Department of Pathology, the Second Xiangya Hospital, Central South University, Changsha 410011, China.

**Keywords:** NSUN2, TP53, RAD51, UCHL3, nasopharyngeal carcinoma, DNA damage

## Abstract

Radiotherapy is the primary treatment for nasopharyngeal carcinoma (NPC), yet radioresistance frequently develops and leads to the failure of treatment for NPC. NSUN2 acts as a potential oncogene in NPC, but its role in NPC radioresistance remains unclear. In this study, we revealed that NSUN2 was upregulated in radioresistant NPC tissues. Through a series of functional assays following radiotherapy, including CCK-8, colony formation, apoptosis analysis by flow cytometry, we demonstrated that NSUN2 promoted radioresistance and enhanced DNA damage repair in NPC cells. Mechanistically, NSUN2 negatively regulated TP53 expression and competitively enhanced the UCHL3-RAD51 interaction, thereby facilitating RAD51 deubiquitination and RAD51-mediated homologous recombination repair of DNA double-strand breaks. Moreover, the suppressive effect of NSUN2 knockdown on NPC radioresistance was reversed by TP53 knockdown. Furthermore, the small molecule GSK-F1 was found to directly bind to NSUN2 and promote its proteasomal degradation, consequently activating the downstream TP53/RAD51 signaling axis and increasing NPC cell cytotoxicity and radiosensitivity. In conclusion, our study elucidates that NSUN2 promotes NPC radioresistance by negatively regulating the TP53/RAD51 axis, and the NSUN2 inhibitor GSK-F1 functions as a radiosensitizer in NPC by disrupting the NSUN2/TP53/RAD51 signaling pathway, thereby providing a potential clinical strategy for the targeted therapy and radiosensitivity in NPC.

## Introduction

Nasopharyngeal carcinoma (NPC) is one of the most common malignant tumors in southern China 1. Ionizing radiation (IR) serves as the cornerstone treatment modality for NPC 2, 3. However, the frequent acquisition of radioresistance in NPC cells often leads to tumor recurrence or metastasis, resulting in poor patient prognosis 4-6. Consequently, elucidating the mechanisms underlying radioresistance, developing radiosensitization strategies, and discovering targeted therapeutics for NPC represent critical and actively pursued scientific challenges in both basic and clinical NPC research.

NOP2/Sun RNA methyltransferase 2 (NSUN2), a principal methyltransferase responsible for 5-methylcytosine (m5C) RNA modification 7, 8, promotes mRNA stability of various targets—including SLC7A11, LINC00324, TEAD1, autotaxin, and TREX2. This activity drives malignant progression and metastasis in multiple cancers, such as endometrial cancer, glioma, esophageal cancer, and bladder cancer 9-15. Our preliminary studies demonstrated that NSUN2 negatively regulates TP53 mRNA stability in an m5C-dependent manner to promote tumor progression of NPC 16, but the function of the NSUN2/TP53 axis in the radioresistance of NPC remains unclear. TP53, a key transcription factor involved in DNA damage repair and apoptosis, influences tumorigenesis by regulating downstream genes such as Bcl-2, TREX1, and TLR8 17-22. It is considered a pivotal determinant of cellular radiosensitivity 23. During the G0/G1 cell cycle checkpoint, TP53 facilitates the detection and repair of DNA damage; if repair fails, TP53 initiates apoptosis. Hence, TP53 plays an essential role in cellular DNA damage response, and its aberrant expression can contribute to radioresistance in tumor cells 21, 23. Therefore, NSUN2 might promote NPC radioresistance through the negative regulation of TP53.

Given the potential significance of NSUN2 in NPC radioresistance, developing NSUN2-targeted therapeutics to improve radiotherapy efficacy represents an urgent clinical need. Small-molecule drugs have gained considerable attention in oncology research due to their advantages over conventional chemotherapeutic agents, including high specificity, reduced toxicity, and favorable oral bioavailability. Notable progress has been made in this area, exemplified by EGFR inhibitors such as gefitinib and erlotinib, and ALK inhibitors like crizotinib and alectinib, which are used in non-small cell lung cancer treatment 24. However, small-molecule inhibitors targeting NSUN2 remain largely unreported.

This study identified high expression of NSUN2 in radioresistant NPC tissues and cell lines. Mechanistically, NSUN2 negatively regulates TP53 expression and competitively enhances the interaction between the deubiquitinase UCHL3 and RAD51, thus promoting RAD51 deubiquitination and protein stabilization, thereby facilitating RAD51-mediated homologous recombination (HR) repair of DNA double-strand breaks and ultimately contributing to NPC radioresistance. Furthermore, using computer-aided molecular docking, we identified a small molecule, GSK-F1, from a chemical library that specifically binds to NSUN2 and promotes its degradation. GSK-F1 exerts antitumor and radiosensitizing effects in NPC by disrupting the NSUN2/TP53/RAD51 signaling axis. These findings offer a promising clinical strategy for targeted therapy and radiotherapy sensitization in NPC.

## Materials and Methods

### Clinical samples and cell culture

A total of 47 nasopharyngeal tissues and 99 NPC tissue samples were obtained from Central South University (SBQLL-2025-091). Among the NPC tissues, twenty-four samples were classified as radiosensitive tissues and nineteen samples as radioresistant tissues. All tissue samples were obtained for analysis following patient consent and pathological evaluation. Cell lines CNE2 and 5-8F, acquired from the Cell Center of Central South University, were cultured in RPMI-1640 and DMEM medium at incubator. (Life Technologies, Carlsbad, CA, USA).

### Plasmids and stable cell lines

Lentiviral infection was employed to generate NPC stable cell lines with knocked-down NSUN2 expression (shNSUN2). A plasmid for TP53 knockdown (shTP53) was also constructed. This study employed the silencing and knockdown sequences were detailed in Table S1.

### Western blot analysis

CNE2 and 5-8F were harvested and lysed with RIPA buffer (New Cell & Molecular Biotech, Suzhou, China) supplemented with a protease inhibitor cocktail for 30 min, with vortexing every 10 min. After quantifying protein concentration, the lysates were denatured for 10 min and subjected to SDS-PAGE and subsequent wet transfer onto PVDF membranes. After blocking, the membranes were incubated with primary antibodies overnight at 4 °C. The primary antibodies used were as follows: GAPDH (1:5,000, Proteintech), NSUN2 (1:2,000, Proteintech, Wuhan, China), γ-H2AX (1:1,000, ABclonal, Wuhan, China), RAD51 (1:1,000, Proteintech), TP53 (1:2,000, Proteintech), UCHL3 (1:1,000, Proteintech), Ubiquitin (Ub, 1:1,000, Santa Cruz, Dallas, TX, USA). After washing with TBST, the membranes were incubated with HRP-conjugated secondary antibodies (Goat Anti-Mouse IgG HCS or Goat Anti-Rabbit IgG HCS, 1:2,000, Abbkine, Wuhan, China) for 1 h at 37 °C. Enhanced chemiluminescence was used to detected protein.

### Cell counting kit-8 (CCK-8) assay

Transfected NPC cells (2,000-3,000 cells in 100 μL medium per well) were seeded into ninety-six-well plates, with five replicate wells per group. After cell attachment, the cells were subjected to irradiation. Then, CCK-8 reagent (Selleck Chemicals, Houston, TX, USA) was added to each well, and the plates were incubated in the dark at 37 °C for 2 h. The absorbance at 450 nm was measured using a microplate reader. This process, including irradiation where applicable, was repeated over a total culture period of 5 days. Dose-response curves were generated using GraphPad Prism 8.0 (GraphPad Software, La Jolla, CA, USA).

### Colony formation assays

NPC cells were trypsinized after transfection, and 1,500-2,000 cells were seeded into twelve-well or six-well plates. After adherence, cells were irradiated and then returned to the incubator for continued culture. When visible clones had formed, the cells were washed two to three times with PBS, fixed with 4% paraformaldehyde, washed again with PBS, and stained with 0.1% crystal violet (Beyotime, Beijing, China). Excess stain was removed by rinsing with distilled water. The plates were scanned, and the number of colonies was counted manually or using image analysis software.

### Apoptosis assay with flow cytometry

Twenty-four hours after transfection, NPC cells were irradiated and cultured for an additional 24 hours. The culture supernatant was collected, and the adherent cells were trypsinized and combined with the supernatant. After centrifugation, the cell pellet was washed once or twice with cold 1 × PBS and resuspended in 1 × binding buffer. The cell suspension was then stained with 5 μL of Annexin V and 5 μL of propidium iodide for 15-20 min in the dark. Apoptosis was analyzed immediately using a flow cytometer (Cytek DxP Athena). Each experiment was independently repeated three times. Data analysis was performed using the FlowJo software (FlowJo LLC, Ashland, OR, USA).

### Immunofluorescence assay

Cells were seeded onto glass coverslips placed in six-well plates. After treatment, cells were fixed with 4% paraformaldehyde for 15 min and permeabilized with 0.3% Triton X-100 for 30 min at room temperature. Non-specific binding sites were blocked with 10% goat serum for 30 min. The cells were then incubated with primary antibody overnight at 4 °C. After washing, the cells were incubated with fluorochrome-conjugated secondary antibody for 1 h at 37 °C in the dark. Nuclei were counterstained with DAPI for 3-5 min in the dark, followed by three washes with 1 × PBS. The coverslips were mounted onto glass slides using an anti-fade mounting medium and imaged using a fluorescence microscope.

### Co-immunoprecipitation (Co-IP) and ubiquitination assay

Protein A/G magnetic beads were washed three times with 1 × PBS. The primary antibody was incubated with the beads for 2-3 hours at room temperature with gentle rotation to form antibody-bead complexes. The complexes were then washed three times. Meanwhile, total cell protein lysates were prepared. A small aliquot of the lysate was saved as the "Input" control. The remaining lysate was equally divided and added to the pre-formed antibody-bead complexes, followed by incubation overnight at 4 °C with rotation. The beads were subsequently washed 3-5 times with lysis buffer, and the bound proteins were eluted by boiling in 1 × SDS loading buffer for subsequent western blot analysis.

### Comet assay

Cells were washed with ice-cold 1 × PBS, harvested, and resuspended at a density of 1×10⁶ cells/mL. A comet assay DNA damage detection kit (Cat: KGA240) was used. The first layer, consisting of 1% normal melting point agarose (NMA), was prepared on a microscope slide and allowed to solidify. Then, the cell suspension was mixed with 0.7% low melting point agarose (LMA) and spread over the first layer. After solidification at 4 °C, a third layer of 0.7% LMA was applied. The slides were immersed in pre-chilled Lysis Buffer and lysed at 4 °C for 2 h. Following lysis, the slides were placed in a freshly prepared alkaline electrophoresis solution for 20 min to allow DNA unwinding. Electrophoresis was conducted at 25 V for 30 min at room temperature. The slides were then neutralized by immersing in neutralization buffer (0.4 M Tris-HCl, pH 7.5) three times for 5 min each. Subsequently, DNA was stained with 20 μL of PI per slide for 10 min in the dark. Images were captured using a fluorescence microscope and analyzed with CASP software.

### Cellular thermal shift assay (CETSA)

Cells were treated with 2 μM GSK-F1 or an equivalent volume of DMSO for 24 h. After digestion and collection, the cells were washed twice with 1 × PBS and resuspended in 1 mL 1 × PBS containing protease inhibitors. The cell suspension was aliquoted equally into eight 0.2 mL PCR tubes. The tubes were heated individually at different temperatures for 3 min in a PCR machine, followed by three freeze-thaw cycles using liquid nitrogen and a 37 °C water bath. The samples were then centrifuged at 12,600 rpm for 30 min at 4 °C. The supernatant from each tube was collected, mixed with 5 × SDS loading buffer, denatured for 10 min, and analyzed by western blot to detect NSUN2 protein levels.

### Drug affinity responsive target stability-western blot (DARTS-WB)

Cells were collected and lysed with lysis buffer, followed by centrifugation to collect the supernatant. The lysates were incubated with either DMSO or GSK-F1 at room temperature for 50 min. Subsequently, pronase E was added based on protein concentration, and hydrolysis was allowed to proceed at room temperature for 30 min. The reaction was terminated by adding protease inhibitors and 5 × SDS loading buffer, followed by boiling. The samples were then subjected to SDS-PAGE electrophoresis. Subsequent steps were performed as described for western blot analysis.

### Immunohistochemistry (IHC)

Tissue sections were baked in an oven at 60 °C for 2-3 hours, deparaffinized in xylene twice (10 min each), and rehydrated through a graded ethanol series (90%, 85%, 75%, 50%) and distilled water. Antigen retrieval was performed by heating the slides in 1 × citrate-based antigen retrieval buffer (pH 9.0) using a microwave oven on low power for 20 min. Endogenous peroxidase activity was blocked with 3% hydrogen peroxide, and non-specific binding was blocked with normal serum. The sections were then incubated overnight at 4 °C with the following primary antibodies: anti-NSUN2 (1:1,000, Proteintech), anti-γ-H2AX (1:200, ABclonal), anti-TP53 (1:500, Proteintech), anti-cleaved PARP (1:100, Proteintech), anti-RAD51 (1:300, Proteintech), and anti-Ki67 (1:100, Bioworld). Subsequently, the sections were incubated with biotin-labeled secondary antibody, followed by streptavidin-horseradish peroxidase. Color development was performed using DAB substrate, and the nuclei were counterstained with hematoxylin. The slides were dehydrated, cleared, mounted, and imaged. Staining was evaluated based on the percentage of positive tumor cells (1: < 25% positive cells, 2: 26-50% positive cells, 3: 51-75% positive cells, 4: > 75% positive cells) and staining intensity (0: no brown particulate staining, 1: light brown particulate staining, 2: moderate brown particulate staining, 3: dark brown particulate staining). A final score < 5 was defined as low expression, and a score ≥ 6 was defined as high expression 25, 26.

### Nude mouse xenograft model

All animal experiments were approved by the Ethics Committee of Central South University (Approval No. APU-2025-0340). Four-week-old female BALB/c nude mice were purchased from Hunan Slake Jingda Experimental Animal Co., Ltd. and housed under specific pathogen-free (SPF) conditions. Stable transfected or control NPC cells (4×10⁶) were subcutaneously injected into the flanks of the mice. When the tumor volume reached approximately 50 mm³, the mice were randomly divided into groups and subjected to local irradiation (6 Gy per dose) and/or treatment with GSK-F1 (5 mg/kg, TargetMol, T19840) via intraperitoneal injection five times. Tumor dimensions (length and width) and mouse body weight were measured and recorded regularly. Tumor volume was calculated using the formula: Volume = (length × width²) / 2. When the tumor volume reached approximately 1,000 mm³, the mice were euthanized humanely in accordance with ethical guidelines, and the tumors were excised and weighed. The harvested tumor tissues were fixed in 4% paraformaldehyde for paraffin embedding, sectioning, and hematoxylin and eosin (H&E) staining.

### Statistical analysis

All data were analyzed using GraphPad Prism 8.0 and are presented as the mean ± standard deviation (SD) or mean ± standard error of the mean (SEM). Comparisons between two groups were performed using an unpaired, two-tailed Student's t-test. Comparisons among multiple groups were analyzed by one-way analysis of variance (ANOVA). A *P*-value of less than 0.05 was considered statistically significant (*P < 0.05, **P < 0.01, ***P < 0.001; ns, no significance).

## Results

### NSUN2 is highly expressed in radioresistant NPC tissues and promotes radioresistance in NPC cells

**Figure 1:**
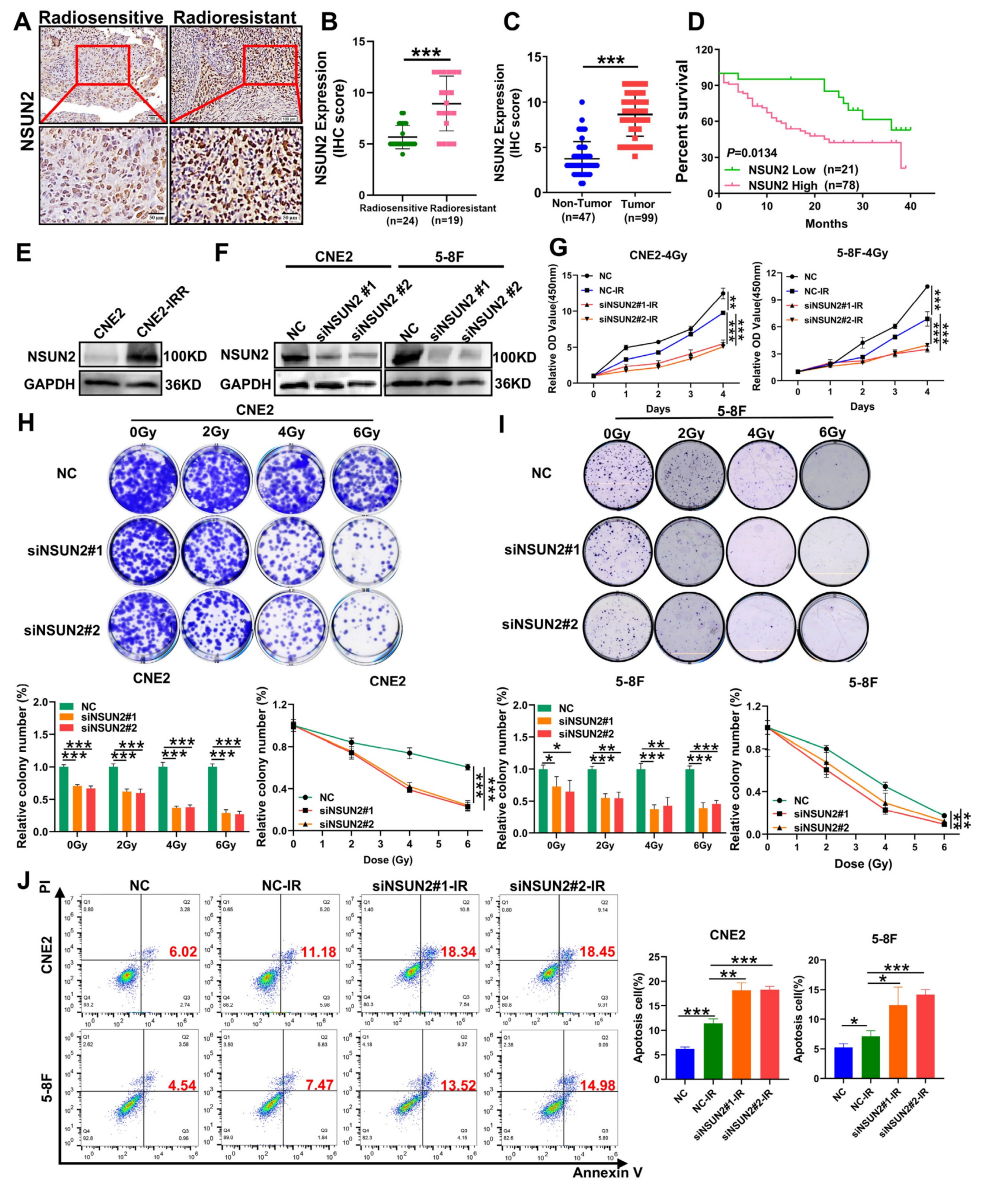
** NSUN2 is highly expressed in radioresistant NPC tissues and promotes radioresistance.** A. IHC assay showing NSUN2 expression in radiotherapy sensitive and radiotherapy resistance NPC tissues. B. Statistical analysis of NSUN2 expression levels in radiotherapy sensitive and radiotherapy resistance NPC tissues. C. Quantification of NSUN2 expression in normal nasopharyngeal tissues and NPC tissues. D. Prognostic analysis of NSUN2 expression in NPC patients. E. NSUN2 protein levels in the parental CNE2 and the derived radioresistant CNE2-IRR. F. Western blot analysis confirming the silence efficiency of NSUN2 in CNE2 and 5-8F cells. G. CCK-8 assay assessing the proliferation of CNE2 and 5-8F cells with NSUN2 knockdown after irradiation. H. Clonogenic assay evaluating the proliferation of NSUN2 knockdown in CNE2 after irradiation. I. Colony formation assay assessing the proliferation of NSUN2 knockdown in 5-8F after irradiation. J. Flow cytometry analysis of apoptosis in CNE2 and 5-8F cells with NSUN2 knockdown after radiotherapy. *: *P* < 0.05; **: *P*<0.01; ***: *P* < 0.001.

Our previous research has confirmed that NSUN2, a key m5C methyltransferase, as a contributor to the malignant progression of NPC 27. To investigate the role of NSUN2 in NPC radioresistance, we performed IHC to detect the differential expression of NSUN2 between the radiotherapy-sensitive (n = 24) and radiotherapy-resistant (n = 19) NPC tissues. As a result, NSUN2 expression was significantly higher in the radioresistant group than the radiosensitive one (Figure 1A, B. Table S2). Furthermore, NSUN2 protein levels were markedly elevated in NPC tissues compared to normal nasopharyngeal tissues, and high NSUN2 expression correlated with poor patient prognosis (Figure 1C, D). Further univariate and multivariate analyses revealed that high NSUN2 expression served as an independent prognostic factor for poor outcomes in the available radiotherapy cohort (Table S3, Table S4). Moreover, we also established a radioresistant NPC cell line, CNE2-IRR, and found that NSUN2 expression was also significantly upregulated in these cells (Figure 1E). These findings suggest an association between NSUN2 and radioresistance. To further explore the functional role of NSUN2 in NPC radioresistance, we generated stable NSUN2-knockdown models in CNE2 and 5-8F NPC cells (Figure 1F). Using CCK-8 and colony formation assays under inducement of various radiation doses (0, 2, 4, and 6 Gy), we assessed the effect of NSUN2 on cell viability. As expected, irradiation reduced NPC cell numbers. Notably, NSUN2 knockdown significantly decreased cell survival rates and the number of colonies formed post-irradiation (Figure 1G-I). Flow cytometric analysis of apoptosis further revealed that silencing NSUN2 increased the rate of radiation-induced apoptosis in NPC cells (Figure 1J). These results indicate that NSUN2 knockdown enhances the inhibitory effects of radiotherapy on cell proliferation and clonogenicity, while promoting apoptosis.

### NSUN2 promotes radiation-induced DNA damage repair by HR-mediated pathway in NPC cells

**Figure 2:**
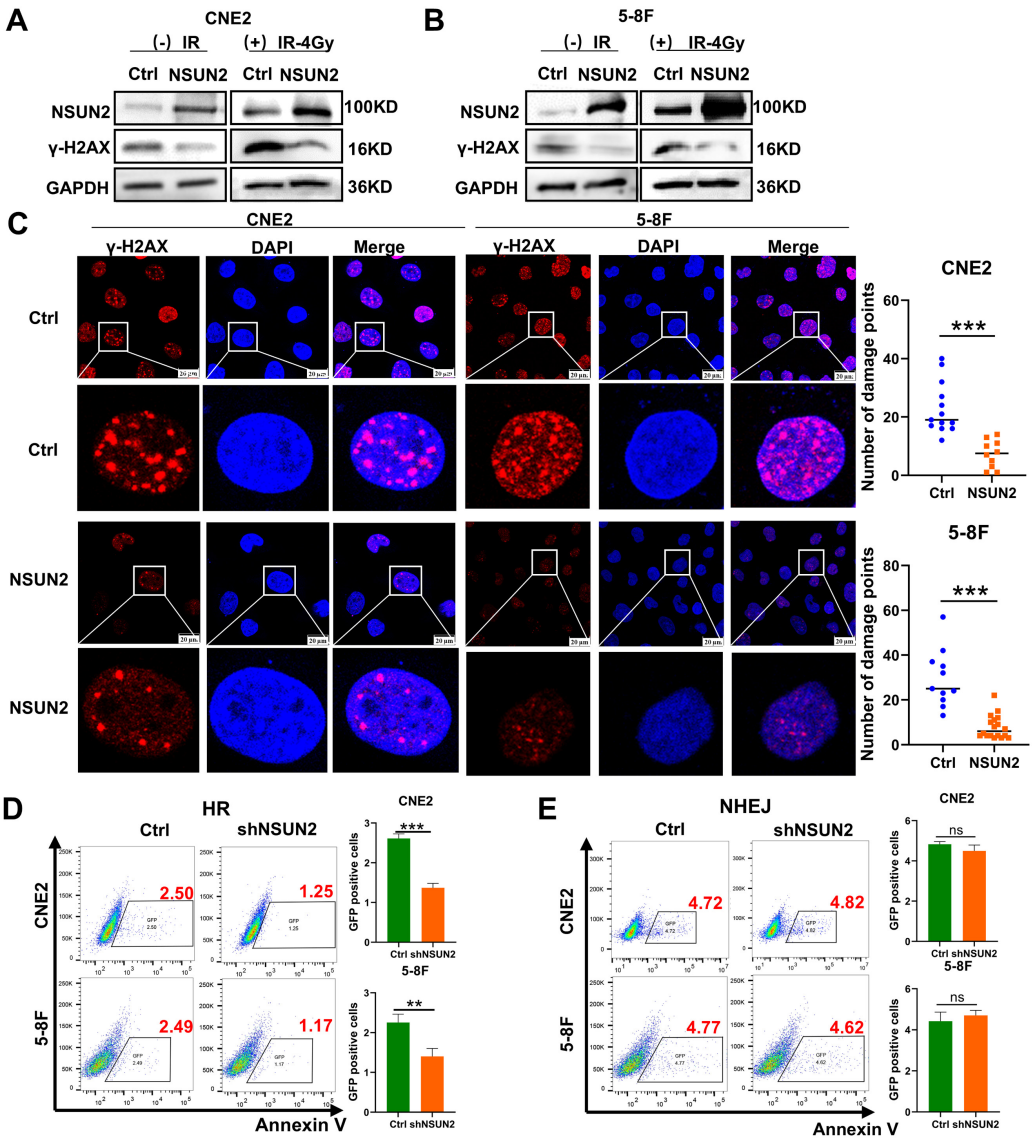
** NSUN2 promotes radiation-induced DNA damage repair in NPC cells.** A. The effect of NSUN2 on γ-H2AX expression was detected by western blot in CNE2. B. The effect of NSUN2 on γ-H2AX expression was detected by western blot in 5-8F. C. Immunofluorescence analysis of γ-H2AX foci in cells overexpressing NSUN2. D. Flow cytometric analysis of HR repair efficiency upon NSUN2 knockdown. E. Flow cytometric analysis of NHEJ repair efficiency upon NSUN2 knockdown. **: *P*<0.01; ***: *P* < 0.001.

Our previous research based on gene set enrichment analysis (GSEA) indicated a strong association between NSUN2 and DNA damage repair pathways 27. As the expression of γ-H2AX will be promoted during the process of DNA double-strand breaks (DSBs) and damage, γ-H2AX can be used as a marker of DNA damage. Therefore, the western blot analysis results showed that irradiation induced γ-H2AX expression, while NSUN2 overexpression significantly reduced γ-H2AX levels (Figure 2A, B). Consistently, immunofluorescence staining demonstrated that NSUN2 overexpression markedly decreased the number of γ-H2AX foci (Figure 2C). DSB repair primarily occurs through HR or non-homologous end joining (NHEJ). To determine which pathway NSUN2 influences, we utilized NSUN2-knockdown NPC cell lines transfected with two distinct DSB repair reporter systems (DR-GFP for HR and EJ5-GFP for NHEJ). Flow cytometry analysis of GFP-positive cells showed that NSUN2 knockdown reduced the number of cells positive for HR-mediated repair, but had no significant effect on NHEJ-mediated repair (Figure 2D, E). These results suggest that NSUN2 deficiency impairs the efficiency of HR-mediated DNA damage repair.

### NSUN2 promotes the protein stability of RAD51 by decreasing the expression of TP53 thereby competitively facilitating the combination of UCHL3 and RAD51

**Figure 3:**
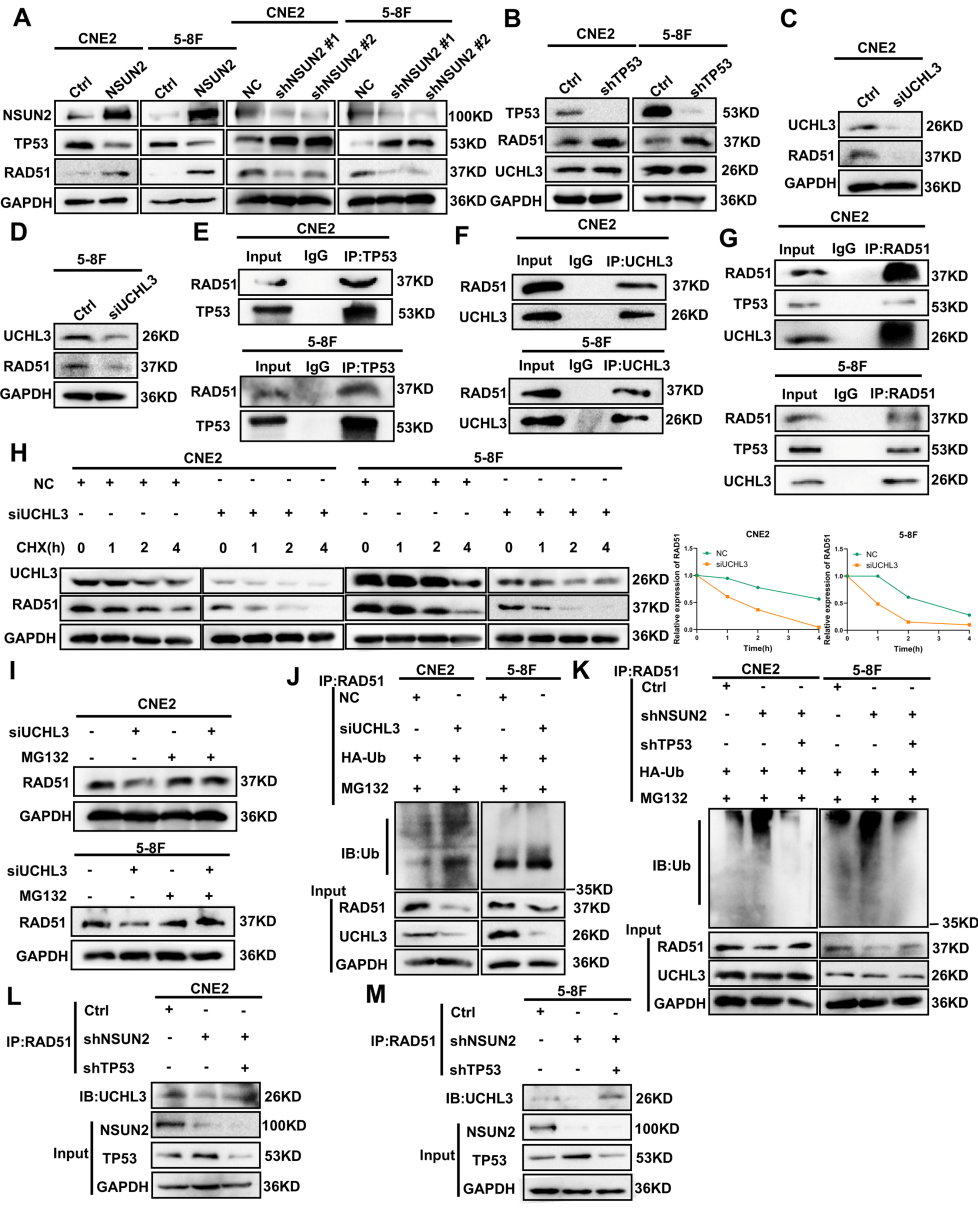
** NSUN2 promotes the protein stability of RAD51 by decreasing the expression of TP53 thereby competitively facilitating the combination of UCHL3 and RAD51.** A. Western blot analysis of TP53 and RAD51 expression in CNE2 and 5-8F cells with NSUN2 overexpression or knockdown. B. Western blot analysis of RAD51 and UCHL3 expression following TP53 knockdown. C. Western blot analysis of RAD51 expression upon UCHL3 silencing in CNE2 cells. D. Western blot analysis of RAD51 expression upon UCHL3 silencing in 5-8F cells. E. Co-IP assay detecting the interaction between TP53 and RAD51. F. Co-IP assay detecting the interaction between UCHL3 and RAD51. G. Co-IP assay detecting the binding of RAD51 to UCHL3 and TP53. H. Western blot analysis of RAD51 protein stability upon UCHL3 silencing. I. Western blot analysis of the mechanism by which UCHL3 regulates RAD51. J. Ubiquitination assay assessing RAD51 ubiquitination levels upon UCHL3 silencing. K. Ubiquitination assay assessing RAD51 ubiquitination levels upon NSUN2 knockdown and TP53 knockdown. L. Co-IP assay evaluating the amount of UCHL3 bound to RAD51 upon NSUN2 knockdown and TP53 knockdown in CNE2 cells. M. Co-IP assay evaluating the amount of UCHL3 bound to RAD51 upon NSUN2 knockdown and TP53 knockdown in 5-8F cells.

Our prior research demonstrated that NSUN2 negatively regulates TP53 mRNA stability via m5C modification, leading to reduced TP53 expression 16. Given that NSUN2 promotes HR-mediated DNA repair and RAD51 is a key factor in HR, we investigated this connection. Firstly, the results of western blot analysis in CNE2 and 5-8F cells showed that NSUN2 overexpression suppressed TP53 protein expression and enhanced RAD51 expression. Conversely, NSUN2 knockdown increased TP53 and decreased RAD51 levels (Figure 3A). As UCHL3 has been reported as a deubiquitinase for RAD51 28, in order to explore whether TP53 competitively inhibits the binding between UCHL3 and RAD51, thus affecting the expression of RAD51, we found that knocking down TP53 can significantly promote the expression of RAD51, but has no effect on the expression of UCHL3 (Figure 3B). Meanwhile, silencing UCHL3 led to a marked decrease in RAD51 expression (Figure 3C, D), and has no effect on the expression of TP53 (Figure S1A). In addition, Co-IP assay results confirmed an interaction between TP53 and RAD51 proteins in NPC cells (Figure 3E). Further Co-IP experiments verified that UCHL3 binds to RAD51 (Figure 3F), and RAD51 can interact with both TP53 and UCHL3 (Figure 3G). Protein stability assays revealed that UCHL3 silencing significantly reduced the half-life of RAD51 protein (Figure 3H). We further explored the way that UCHL3 regulates RAD51, and we found that UCHL3 promotes the expression of RAD51 through ubiquitin proteasome pathway, not autophagy lysosome by treatment with the proteasome inhibitor MG132 and autophagy-lysosome inhibitor chloroquine (CQ) (Figure 3I, Figure S1B, C). This indicates that UCHL3 regulates RAD51 stability primarily via the ubiquitin-proteasome pathway, suggesting UCHL3 acts as a key deubiquitinase for RAD51 in NPC.

Additionally, ubiquitination assays further confirmed that UCHL3 silencing enhanced RAD51 ubiquitination in both CNE2 and 5-8F cells (Figure 3J). To determine if NSUN2 affects RAD51 ubiquitination by modulating the competitive binding of TP53 and UCHL3 to RAD51, we performed a reversal experiment. NSUN2 knockdown elevated RAD51 ubiquitination, TP53 knockdown counteracted this increase. (Figure 3K). At the same time, Co-IP assays demonstrated that NSUN2 knockdown reduced the amount of UCHL3 bound to RAD51, while TP53 knockdown promoted the interaction between UCHL3 and RAD51 (Figure 3L, M).

### NSUN2 promotes DNA damage repair and radioresistance in NPC cells by negatively regulating TP53/RAD51 axis

**Figure 4:**
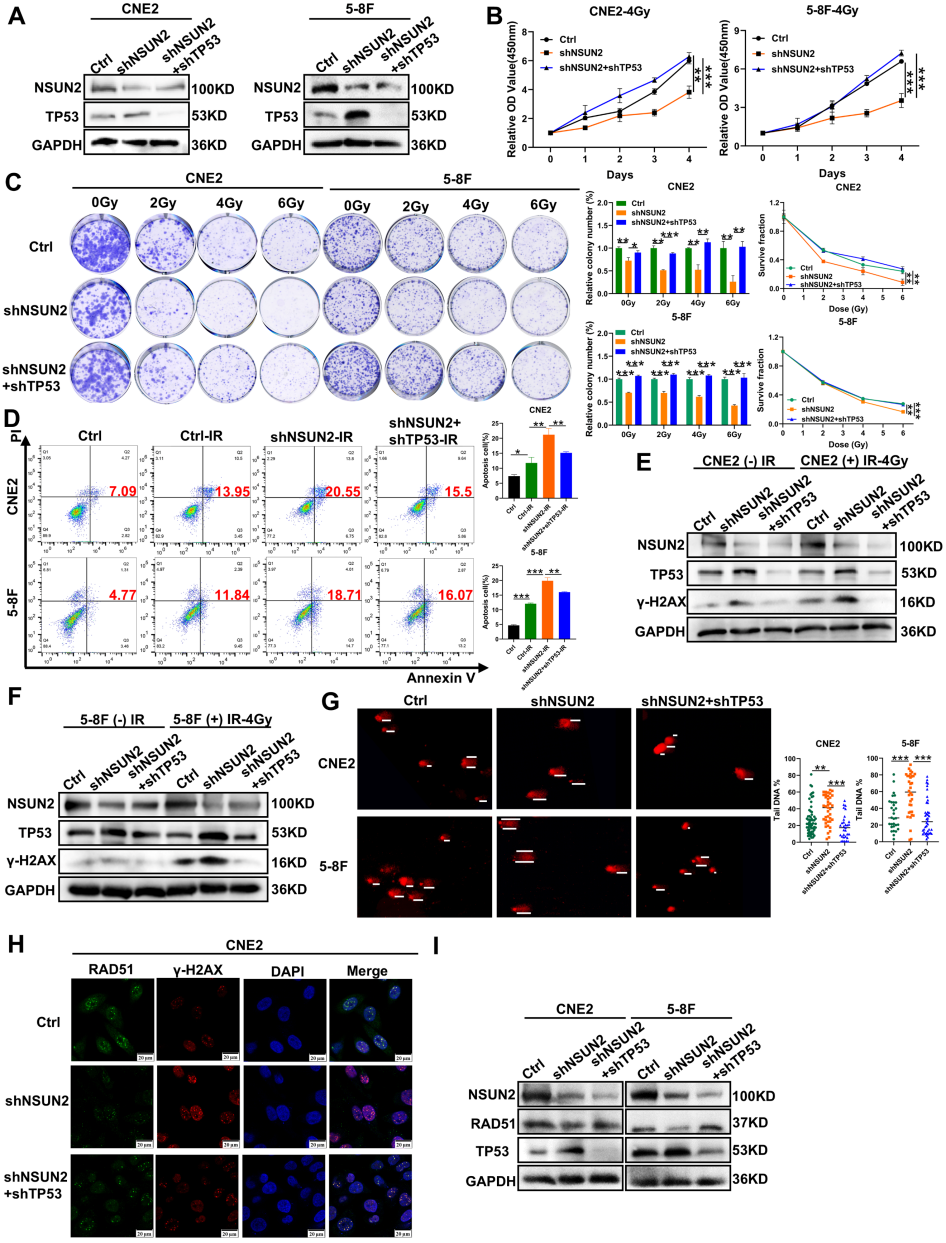
** NSUN2 promotes radiation-induced radioresistance and DNA damage repair in NPC cells by negatively regulating TP53/RAD51 axis.** A. Western blot analysis of TP53 reversal efficiency in CNE2 and 5-8F cells. B. CCK-8 assay evaluating cell proliferation after irradiation in CNE2 and 5-8F cells with NSUN2 and TP53 knockdown. C. Colony formation assay assessing clonogenic ability after irradiation in cells with NSUN2 and TP53 knockdown. D. Flow cytometric analysis of apoptosis after radiotherapy in cells with NSUN2 and TP53 knockdown. E. Western blot analysis of γ-H2AX expression in CNE2 cells with TP53 reversal after irradiation. F. Western blot analysis of γ-H2AX expression in 5-8F cells with TP53 reversal after irradiation. G. Comet assay measuring DNA migration with TP53 reversal after irradiation. H. Immunofluorescence staining showing RAD51 and γ-H2AX expression in CNE2 cells with TP53 reversal. I. Western blot was used to detect the effect of reversing TP53 on RAD51 expression. **: *P*<0.01; ***: *P* < 0.001.

To investigate whether NSUN2 promotes radiation-induced DNA damage repair by negatively regulating TP53, we first confirmed that NSUN2 knockdown increased TP53 levels via western blot assay in CNE2 and 5-8F cells, and further reversing TP53 also indicated that the expression of TP53 was effectively reduced (Figure 4A). CCK-8 and colony formation assays combined with radiation irradiation demonstrated that NSUN2 knockdown significantly reduced cell proliferation and colony formation after radiation, while knocking down TP53 expression can reverse the proliferation and clone formation of NPC cells (Figure 4B, C). Flow cytometry for apoptosis revealed that NSUN2 silencing increased radiation-induced apoptosis, whereas TP53 knockdown counteracted this pro-apoptotic effect (Figure 4D).

Western blot analysis revealed low basal γ-H2AX expression in non-irradiated cells. Radiation induced γ-H2AX expression, which was further enhanced by NSUN2 knockdown. This NSUN2 knockdown-induced increase in γ-H2AX was attenuated by TP53 knockdown (Figure 4E, F). Meanwhile, the comet assay revealed that NSUN2 knockdown increased DNA tail migration following irradiation, indicating enhanced DNA damage. This effect was reversed by TP53 knockdown, which reduced DNA migration and promoted repair (Figure 4G). In addition, immunofluorescence staining for RAD51, a key molecule in HR repair, and γ-H2AX, a DNA damage molecule, in NPC cells revealed that NSUN2 knockdown increased γ-H2AX foci formation (more DNA damage) and decreased the aggregation of RAD51. Crucially, TP53 knockdown in the NSUN2-knockdown background reversed the aggregation of RAD51 at damage sites and reduced the expression of γ-H2AX, thus promoting DNA repair (Figure 4H, Figure S2A). RAD51 expression was also elevated in the radioresistant CNE2-IRR cells during DNA damage response (Figure S2B). Finally, western blot analysis confirmed that NSUN2 knockdown reduced RAD51 protein levels, and this reduction was restored by TP53 knockdown (Figure 4I). These findings suggest that NSUN2 negatively regulates the expression of TP53 to enhance RAD51 protein stability via the ubiquitin-proteasome pathway, thereby promoting radiation-induced HR repair and reducing radiosensitivity in NPC cells.

### *In vivo* validation that NSUN2 promotes NPC radioresistance by negatively regulating TP53/RAD51 axis

**Figure 5:**
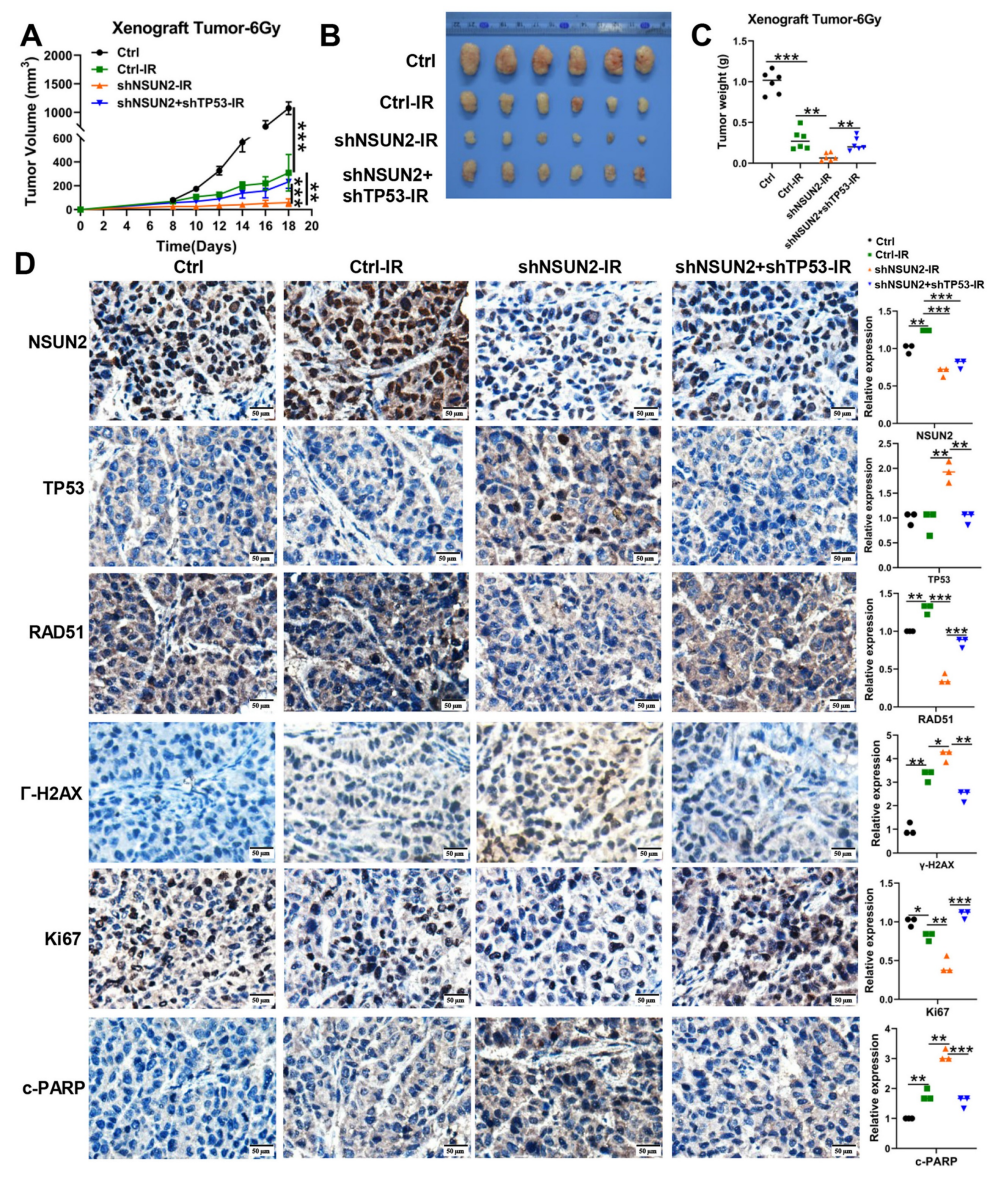
***In vivo* validation that NSUN2 promotes NPC radioresistance by negatively regulating TP53/RAD51 axis.** A. Tumor growth curves of xenografts. B. The images of tumor. C. Quantitative analysis of tumor weights. D. The expression of NSUN2, TP53, RAD51, γ-H2AX, Ki67 and c-PARP in tumor tissues of mice was detected by IHC (higher power view). *: *P* < 0.05; **: *P*<0.01; ***: *P* < 0.001.

To evaluate the impact of NSUN2 on radioresistance *in vivo* and the role of TP53, we subcutaneously injected nude mice with stable CNE2 control (shPLVX), NSUN2-knockdown (shNSUN2), or shNSUN2+shTP53 cells. As expected, radiotherapy significantly inhibited tumor growth compared to the control group (Figure S3A, Figure 5A). NSUN2 knockdown combined with irradiation potently suppressed tumor growth. Importantly, TP53 knockdown significantly reversed this tumor growth inhibition caused by NSUN2 knockdown (Figure 5B, C), without affecting mouse body weight (Figure S3B).

IHC analysis of mouse xenografts for NSUN2, TP53, RAD51, γ-H2AX, the proliferation marker Ki67 and the apoptosis marker cleaved-PARP (c-PARP) revealed that NSUN2 knockdown increased TP53, γ-H2AX and c-PARP expression, decreased RAD51 and Ki67 expression. Concurrent TP53 knockdown in the NSUN2-knockdown background reversed these effects, restoring RAD51 and Ki67 expression and reducing c-PARP and γ-H2AX levels (Figure 5D, Figure S3C).

### A novel small-molecule inhibitor GSK-F1 targeting NSUN2 promotes its protein degradation

**Figure 6:**
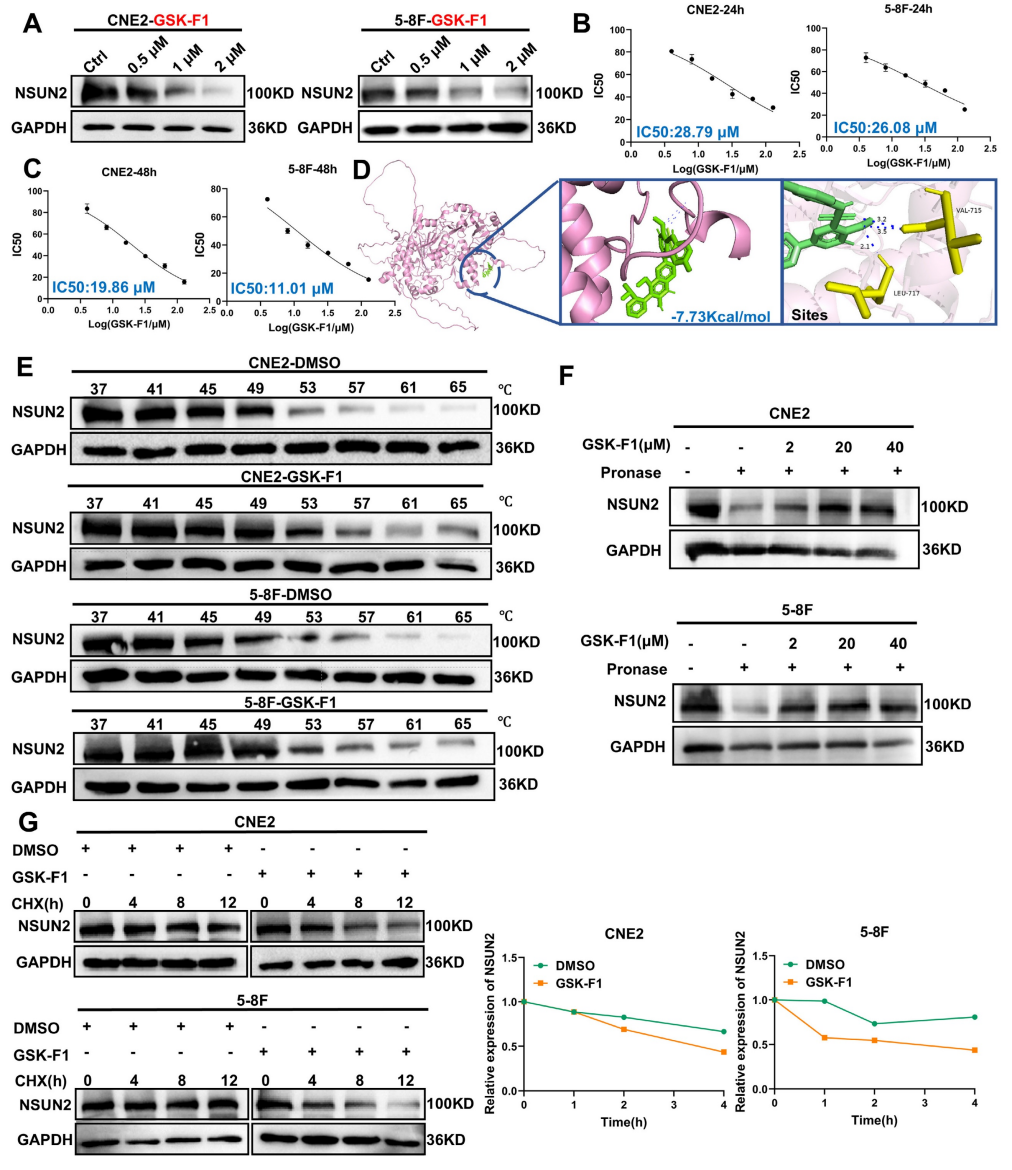
** GSK-F1 binds and reduces the stability of NSUN2 protein.** A. Western blot analysis of NSUN2 protein levels in CNE2 and 5-8F cells treated with GSK-F1. B. CCK-8 assay determining IC50 values of GSK-F1 in CNE2 and 5-8F cells at 24 h. C. CCK-8 assay determining IC50 values of GSK-F1 in CNE2 and 5-8F cells at 48 h. D. Molecular docking simulation showing the binding mode of GSK-F1 with NSUN2. E. The binding of NSUN2 and GSK-F1 in NPC cells was detected by CETSA assay. F. The binding of GSK-F1 and NSUN2 in NPC cells was detected by DARTS-WB. G. Protein stability assay evaluating NSUN2 degradation upon GSK-F1 treatment.

Given that NSUN2 overexpression and the consequent dysregulation of the TP53/RAD51 axis represent a key mechanism underlying radioresistance in NPC, we aimed to identify small-molecule inhibitors targeting NSUN2 as a therapeutic strategy to enhance radiosensitivity. Using computer-aided molecular docking, we screened a library of 16,672 compounds and identified 100 potential NSUN2 inhibitors. Subsequent evaluation based on binding energy and literature review, focusing on unreported anti-tumor properties and drug-like features such as oral bioavailability, narrowed this list to 11 promising candidate inhibitors. Subsequent validation using western blot identified GSK-F1, an orally active compound, as effectively reducing NSUN2 protein levels (Figure 6A, Figure S4). To assess the cytotoxicity of GSK-F1 in NPC cells, we determined its IC50 values using CCK-8 assays. The IC50 for GSK-F1 in CNE2 cells was 28.79 μM at 24 h and 19.86 μM at 48 h. In 5-8F cells, the IC50 was 26.08 μM at 24 h and 11.01 μM at 48 h (Figure 6B, C). Molecular docking with PyMOL suggested that GSK-F1 binds to NSUN2 at residues VAL-715 and LEU-717 (binding energy is -7.73Kcal/mol) (Figure 6D). Cellular thermal shift assay (CETSA) confirmed the direct binding of GSK-F1 to NSUN2 within cells (Figure 6E). DARTS-WB further confirmed that GSK-F1 can bind to NSUN2, thereby conferring resistance to hydrolysis of Pronase E (Figure 6F). Furthermore, protein stability assays demonstrated that GSK-F1 treatment accelerated NSUN2 degradation (Figure 6G).

### GSK-F1 binds to NSUN2 and promotes radiosensitization in NPC cells

**Figure 7:**
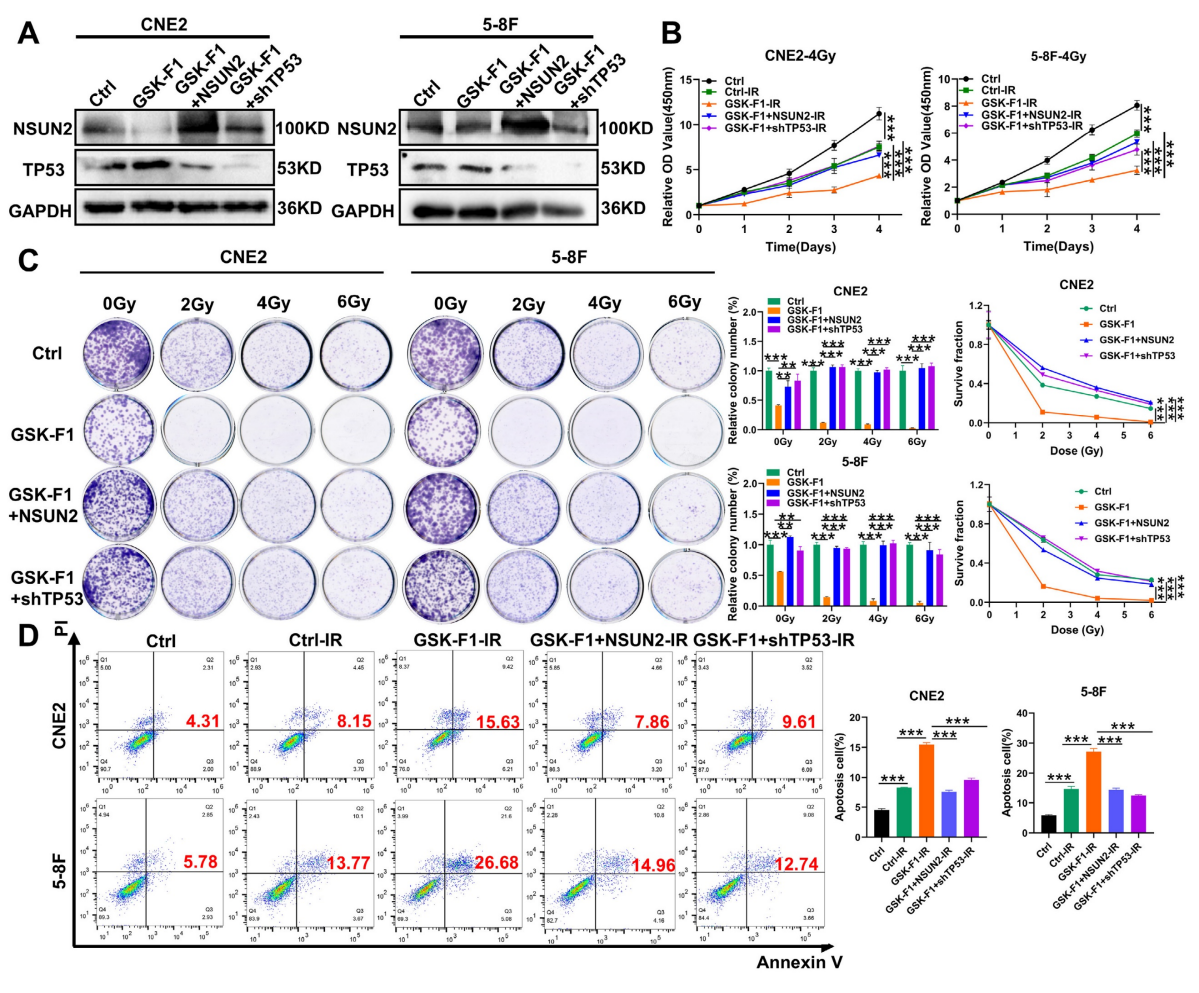
**GSK-F1 targets NSUN2 to promote radiosensitization in NPC cells.** A. Western blot analysis the reversal efficiency of NSUN2 and TP53. B. CCK-8 assay evaluating cell proliferation under irradiation with GSK-F1 treatment and reversal of NSUN2 and TP53 in NPC cells (GSK-F1:4 μM, treatment for 24h). C. Clonal formation assay evaluating cell clonal forming ability under irradiation with GSK-F1 treatment and reversal of NSUN2 and TP53 in NPC cells (GSK-F1:4 μM, treatment for 24h). D. Apoptosis analysis under irradiation with GSK-F1 treatment and reversal of NSUN2 and TP53 in NPC cells. **: *P*<0.01; ***: *P* < 0.001.

To further elucidate the mechanism of GSK-F1 targeting NSUN2 in radiosensitization of NPC, we first confirmed that GSK-F1 treatment reduced NSUN2 levels by western blot assay. Overexpression of NSUN2 or knockdown of TP53 in GSK-F1-treated cells effectively reversed NSUN2 and TP53 expression levels, respectively (Figure 7A). Radiation-induced CCK-8 and colony formation assays indicated that GSK-F1 treatment significantly inhibited cell proliferation and enhanced radiosensitivity. These effects were reversed by either NSUN2 overexpression or TP53 knockdown (Figure 7B, C). Similarly, apoptosis assays demonstrated that the combination of GSK-F1 and irradiation significantly increased apoptosis, and this increase was attenuated by NSUN2 overexpression or TP53 knockdown (Figure 7D).

### *In vivo* validation that GSK-F1 promotes radiosensitization in NPC by negatively regulating NSUN2/TP53/RAD51 axis-mediated HR repair

**Figure 8:**
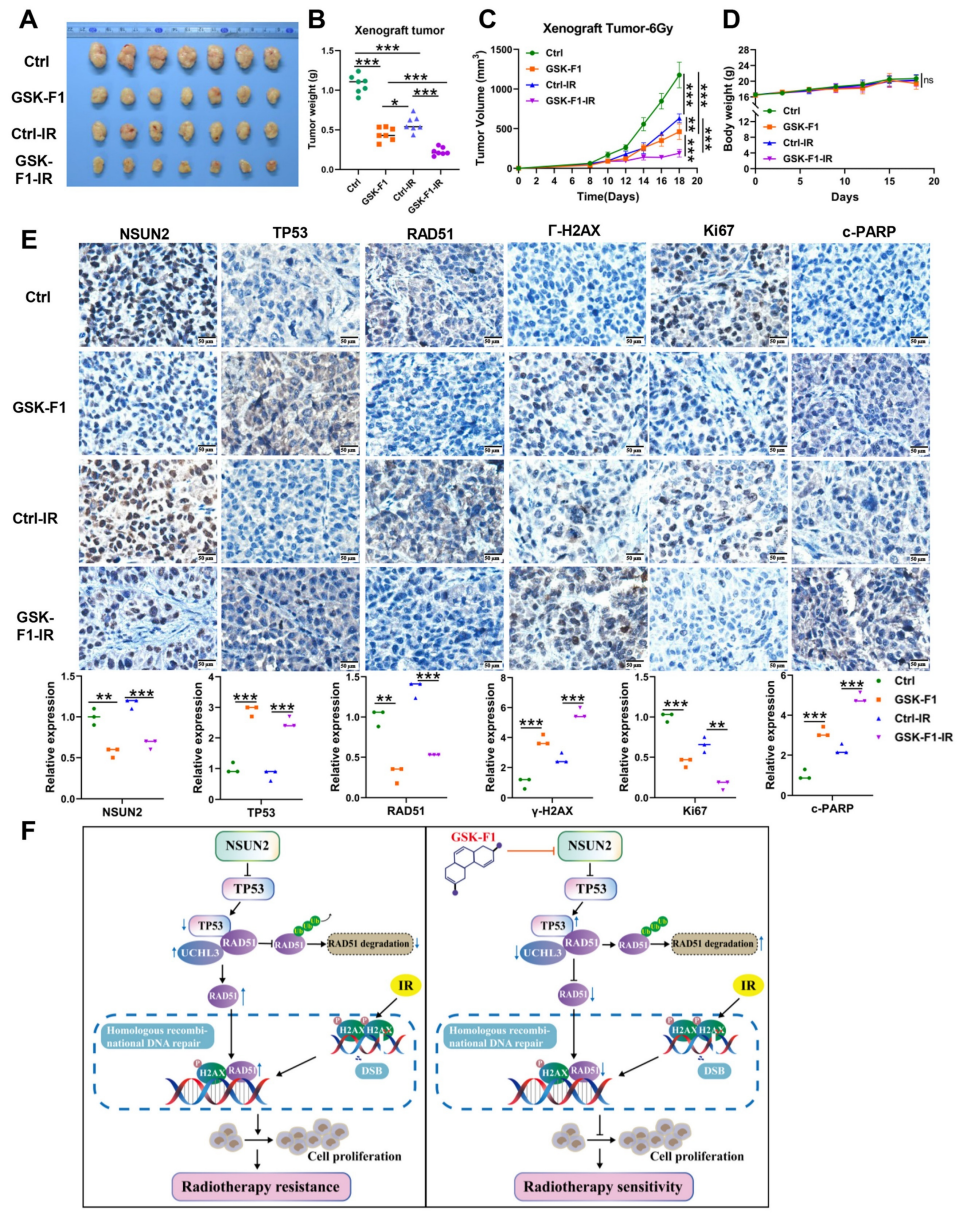
***In vivo* validation that GSK-F1 targets NSUN2 to promote radiosensitization in NPC cells.** A. Representative images of tumor-bearing nude mice. B. Statistics of tumor weight. C. Tumor volume growth curves of xenografts. D. Body weights statistics of mice. E. The expression of NSUN2, TP53, RAD51, γ-H2AX, Ki67 and c-PARP in tumor tissues of mice was detected by IHC (higher power view). F. Schematic model illustrating the mechanism. *: *P* < 0.05; **: *P*<0.01; ***: *P* < 0.001.

To evaluate the function of GSK-F1 on radiosensitization *in vivo*, we established CNE2 xenograft models in mice. Subcutaneous tumor experiments showed that GSK-F1 treatment alone significantly inhibited tumor growth. Furthermore, the combination of GSK-F1 and radiotherapy resulted in a more potent suppression of tumor growth compared to either treatment alone (Figure 8A-C, Figure S5A), with no significant effect on mouse body weight (Figure 8D). IHC analysis of xenograft tissues for NSUN2, TP53, RAD51, γ-H2AX, Ki67, and c-PARP revealed that GSK-F1 treatment downregulated NSUN2, RAD51, and Ki67, while upregulating TP53, γ-H2AX, and c-PARP (Figure 8E, Figure S5B). Western blot analysis confirmed that radiation and GSK-F1 treatment had no effect on UCHL3 expression (Figure S5C). Moreover, Hematoxylin and eosin (H&E) staining of major organs (heart, liver, spleen, lung, and kidney) showed no apparent toxicity upon GSK-F1 treatment (Figure S5D). These results indicate that GSK-F1 effectively targets NSUN2 to promote radiosensitization in NPC, both *in vitro* and *in vivo*. In conclusion, GSK-F1 targets NSUN2, modulates the TP53/RAD51 axis, inhibits DNA damage repair, and consequently promotes radiosensitization in NPC (Figure 8F).

## Discussion

Radiotherapy is a highly effective treatment for early-stage NPC. IR exerts anti-tumor effects by inducing DNA damage in tumor cells, leading to apoptosis or irreversible cell cycle arrest. However, inherent cellular radiation defense mechanisms can repair this DNA damage, influencing tumor radiosensitivity and ultimately leading to the development of radioresistance 29. NSUN2 has been identified as a potential oncogene in NPC progression. Studies have shown that NSUN2 depletion can promote DNA double-strand breaks (DSBs) and compromise genomic integrity 30. For example, NSUN2 deficiency promotes DSBs in pancreatic cancer 31. Consistent with these findings, our study demonstrates that NSUN2 is highly expressed in radioresistant NPC tissues. Silencing NSUN2 inhibited the proliferative and clonogenic capacity post-irradiation and promoted radiation-induced apoptosis. Furthermore, we established that NSUN2 participates in the DSB response pathway. Specifically, NSUN2 reduced the expression of the DNA damage marker γ-H2AX and decreased the formation of γ-H2AX nuclear foci, indicating its role in promoting DNA repair via HR. Indeed, Wu reported a regulatory role for NSUN2 in R-loop dynamics and damage repair in bladder cancer cells. Mechanistically, NSUN2 promotes bladder cancer progression by recruiting EZH2 to facilitate the epigenetic silencing of the tumor suppressor gene PRDM11. Additionally, NSUN2 knockdown sensitized tumors to cisplatin, resulting in reduced tumor growth, increased DNA damage levels, and impaired HR repair by reducing MRE11 recruitment to damage sites 32. Moreover, NSUN2 has been associated with the progression and acquisition of radioresistance 33. However, the specific role of NSUN2 in radiotherapy-induced DSB repair and radioresistance remained unclear. Therefore, our findings are the first to report that NSUN2 promotes the formation of radioresistance in NPC by facilitating DSB repair.

This study demonstrates that TP53 knockdown reverses the suppressive effects of NSUN2 silencing on post-irradiation cell proliferation and clonogenic ability, as well as the promotive effect on apoptosis. Conversely, TP53 knockdown reduced DNA migration length and nuclear foci formation, ultimately promoting radioresistance in NPC cells. TP53 plays pivotal roles in diverse processes such as DNA damage repair 22. TP53 plays a critical role in the DNA damage response and is a key determinant of cellular radiosensitivity. TP53 operates at the G0/G1 cell cycle checkpoint to detect and initiate repair of damaged DNA; if repair fails, TP53 triggers apoptosis. Thus, our results confirm the pivotal role of TP53 in NSUN2-mediated DNA damage response and radioresistance in NPC.

NSUN2 primarily promotes radiation-induced DNA damage repair in NPC cells through HR. RAD51 is an essential factor in this repair process. During HR, the DNA damage site is resected to produce 3' single-stranded DNA (ssDNA) tails. RAD51 then assembles on these tails to form a helical nucleoprotein filament, which mediates strand invasion into the homologous template to create a displacement loop (D-loop). Finally, DNA synthesis and repair are completed with the aid of other HR-associated proteins 34. Meanwhile, RAD51 is an unstable protein, and UCHL3 has been identified as a potential deubiquitinase that stabilizes RAD51 by preventing its degradation 28. In this study, we found that TP53 can bind to RAD51 and negatively regulate its protein expression. We further demonstrated that UCHL3 binds to and stabilizes RAD51 in NPC cells. Crucially, we provide evidence that TP53 competes with UCHL3 for RAD51 binding, thereby antagonizing UCHL3-mediated RAD51 deubiquitination and leading to decreased RAD51 expression. This competition influences radiation-induced DNA damage repair and radiosensitivity. Furthermore, NSUN2, by negatively regulating TP53 expression, promotes UCHL3-mediated deubiquitination and stabilization of RAD51. NSUN2 knockdown reduced RAD51 recruitment to DNA damage sites and its overall expression, effects that were reversed by TP53 knockdown. Therefore, we conclude that NSUN2 promotes radiotherapy-induced DSB repair via HR and NPC radioresistance by modulating the TP53/RAD51 signaling axis.

NSUN2 acts as an oncogene in various cancers, where its high expression may serve as a potential biomarker 11, 13, 35. Consequently, targeting NSUN2 could provide novel therapeutic strategies for NPC and mitigate radioresistance. However, no specific NSUN2 inhibitors have been reported. In this study, we employed molecular docking to screen for potential NSUN2 inhibitors and identified GSK-F1 as a small molecule that significantly suppresses NSUN2 expression and increases cytotoxicity in NPC cells. GSK-F1 demonstrated strong binding affinity for NSUN2 and promoted its degradation. Furthermore, *in vitro* and *in vivo* experiments under irradiation confirmed that GSK-F1 targets NSUN2 to promote radiosensitization in NPC. Small-molecule targeted therapies, known for their higher efficacy and lower toxicity compared to conventional chemotherapy, have made significant strides in oncology research 36. Notable examples include PARP inhibitors like olaparib for BRCA-mutant breast and ovarian cancer. Therefore, our discovery of NSUN2 small-molecule inhibitor offers a promising new therapeutic strategy for the clinical management of NPC.

## Conclusion

In conclusion, our study demonstrates that NSUN2, which is highly expressed in radioresistant NPC tissues, promotes radioresistance and DNA damage repair. Mechanistically, NSUN2 negatively regulates TP53 expression and competitively enhances the UCHL3-RAD51 interaction, thereby facilitating RAD51 deubiquitination and RAD51-mediated HR repair. The radioresistance-inhibiting effect of NSUN2 knockdown was reversed by TP53 knockdown. Furthermore, we identified a novel small molecule inhibitor, GSK-F1, which binds directly to NSUN2, promotes its degradation, and effectively counteracts NSUN2-driven radioresistance in NPC.

## Supplementary Material

Supplementary tables and figures.

## Data Availability

The data that support the findings of this study are available from the corresponding author upon reasonable request.

## Abbreviations

NPC: Nasopharyngeal carcinoma
IR: Ionizing radiation
NSUN2: NOP2/Sun RNA methyltransferase 2
CCK-8: Cell Counting Kit-8
Co-IP: Co-immunoprecipitation
IHC: Immunohistochemistry
DSBs: DNA double-strand breaks
HR: homologous recombination
