# Supplementary material for: A novel small-molecule inhibitor GSK-F1 confers radiosensitivity by inhibiting the NSUN2/TP53/RAD51 axis-mediated DNA homologous recombination repair in nasopharyngeal carcinoma
Source: Int J Biol Sci. 2026 Mar 30;22(8):4043–58. doi: 10.7150/ijbs.130087 (PMC13137861; doi:10.7150/ijbs.130087)
Supplement: Supplementary file 1 — Supplementary tables and figures. [file ijbsv22p4043s1.pdf]

Table S1. Sequences used in this study

Targets	Sequences
siNSUN2#1	5’-GGAGAACAAGCTGTTCGAG-3’
siNSUN2#2	5’-GAGATCCTCTTCTATGATC-3’
siUCHL3	5’-GGCACCAAGUAUAGAUGATT-3’
shNSUN2	F:GATCCGAAGCATCGTGCTGAAGTACTCGAGTACTTCAGCACGATGCTTCTTTTTG
	R:AATTCAAAAAGAAGCATCGTGCTGAAGTACTCGAGTACTTCAGCACGATGCTTCG
shTP53	F:GATCCGACTCCAGTGGTAATCTACCTCGAGGTAGATTACCACTGGAGTCTTTTTG
	R:AATTCAAAAAGACTCCAGTGGTAATCTACCTCGAGGTAGATTACCACTGGAGTCG
Primers	
NSUN2	F: 5’-AAGAAAGATGGCGTGTGTGG-3’
	R: 5’-TATTCAGCAGCACATTCCGC-3’
GAPDH	F: 5’-CAACGGATTTGGTCGTATTGG-3’
	R: 5’-TGACGGTGCCATGGAATTT-3’

Table S2. Correlation between NSUN2 expression and clinicopathologic characters of NPC samples.

Characteristics	NSUN2 expression		*P*
	Low expression	High expression	
Age（year）			
≤52（n=50）	11（22%）	39（78%）	0.8464
＞52（n=49）	10（20.41%）	39（79.59%）	
Gender			
Male（n=73）	13（17.81%）	60（82.19%）	0.1651
Female（n=26）	8（30.77%）	18（69.23%）	
Clinical stages			
I-II（n=32）	17（53.13%）	15（46.87%）	***P < 0.0001
III-IV（n=67）	4（5.97%）	63（94.03%）	
Tumor size			
T1-2（n=43）	18（41.86%）	25（58.14%）	*** P <0.0001
T3-4（n=56）	3（5.36%）	53（94.64%）	
Lymph node metastasis			
No（n=12）	9（75%）	3（25%）	*** P <0.0001
Yes（n=87）	12（13.79%）	75（86.21%）	
Radiosensitivity			
Radiosensitive（n=24）	16（66.67%）	8（33.33%）	** P <0.01
Radioresistant（n=19）	4（21.05%）	15（78.95%）	

Table S3. NSUN2 and clinicopathologic characters in univariate manners.

Characteristics	Category	χ^2	*HR* (95%*CI*)	Log-rank *P*
Age	≤52<br>>52	0.6321	1.251 (0.7101-2.206)	0.4266
Gender	Male<br>Female	0.01995	0.956 (0.509-1.795)	0.8877
Expression of NSUN2	Low<br>High	6.111	2.419 (1.323-4.423)	* P <0.05
Clinical stages	I-II<br>III-IV	5.661	2.178 (1.22-3.887)	* P <0.05
Lymph node metastasis	No<br>Yes	2.746	2.266 (1.069-4.806)	0.0975
Tumor size	T1-2<br>T3-4	20.65	4.237 (2.404-7.467)	*** P <0.0001
Radiosensitivity (n=43)	Radiosensitive<br>Radioresistant	35.33	11.74 (4.617-29.88)	*** P <0.0001

Note: HR: Hazard Ratio; P<0.05 represent statistical differences. Only 43 samples had radiotherapy information.

Table S4. NSUN2 and clinicopathologic characters in multivariate manners.

Characteristics	Group	*HR*	95%*CI*	*P* value
Expression of NSUN2	High vs Low	7.957	1.745-36.278	** *P* <0.01
Clinical stages	III-IV vs I-II	1.365	0.397-4.687	0.6213
Lymph node metastasis	Yes vs No	1.534	0.192-12.25	0.6863
Tumor size	T3-4 vs T1-2	1.454	0.288-7.338	0.6506
Radiosensitivity	Radioresistant vs Radiosensitive	10.139	1.772-58.001	** *P* <0.01

Note: HR: Hazard Ratio; *P*<0.05 represent statistical differences. Multivariate Cox regression analysis was performed based on the sample size of radiosensitivity (n=43).

Supplementary figures

A novel small-molecule inhibitor GSK-F1 confers radiosensitivity by inhibiting the NSUN2/TP53/RAD51 axis-mediated DNA homologous recombination repair in nasopharyngeal carcinoma

Lemei Zheng[1,2,3], Mengna Li[2,3], Xiaolong Li[1,4], Jianxia Wei[1,2,3], Changning Xue[1,2,3], Qingqing Wei[1,2,3], Yumei Duan[1,2,3], Huizhen Xin[1,2,3], Zubing Wu[1,2,3], Ting Zeng[1,2,3], Wei Xiong[1,2,3], Songqing Fan[4], Ming Zhou[1,2,3]*, Hongyu Deng[1,2]*

Figure S1

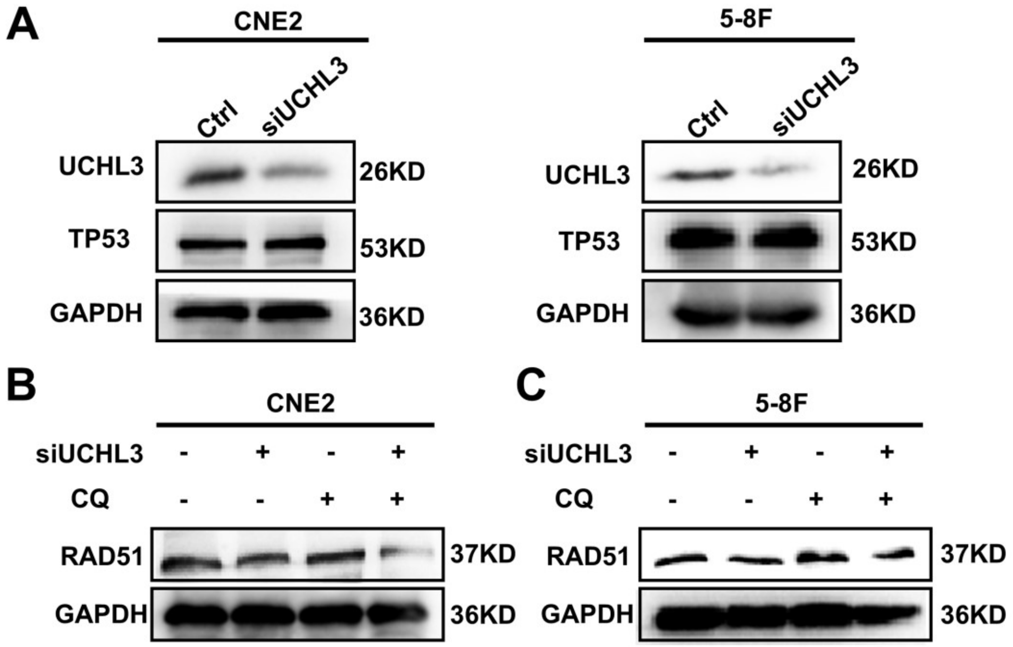

Figure S1. The effect of UCHL3 on TP53 and RAD51 expression. A. The effect of UCHL3 silencing on TP53 expression was detected by Western blot analysis in CNE2 and 5-8F cells. B. Western blot analysis of RAD51 expression in CNE2 ells upon UCHL3 knockdown with CQ treatment. C. Western blot analysis of RAD51 expression in 5-8F cells upon UCHL3 knockdown with CQ treatment.

Figure S2

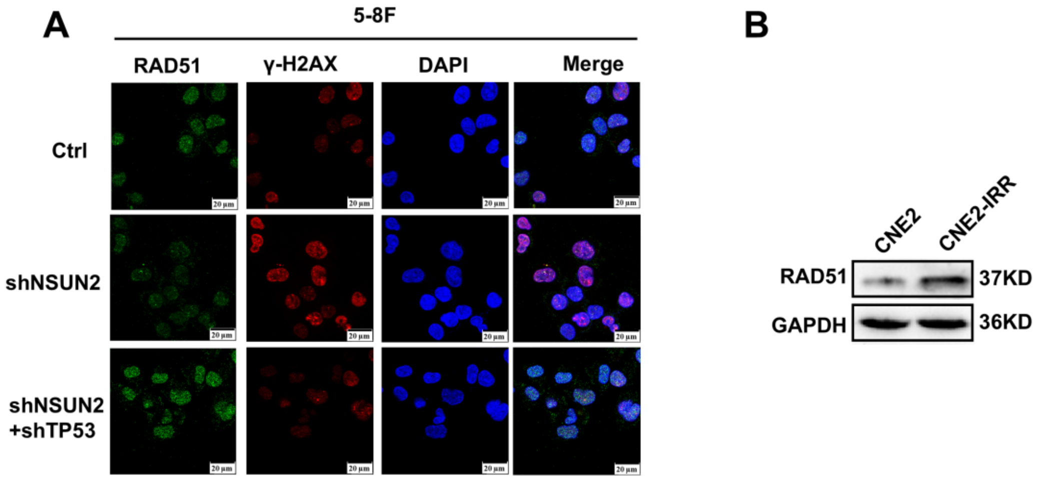

Figure S2. Effects of NSUN2 knockdown and TP53 reverse on DNA damage repair. A. Immunofluorescence assay detecting the expression of RAD51 and γ-H2AX with TP53 reverse in 5-8F cells. B. Western blot analysis of RAD51 expression in CNE2-IRR cells.

Figure S3

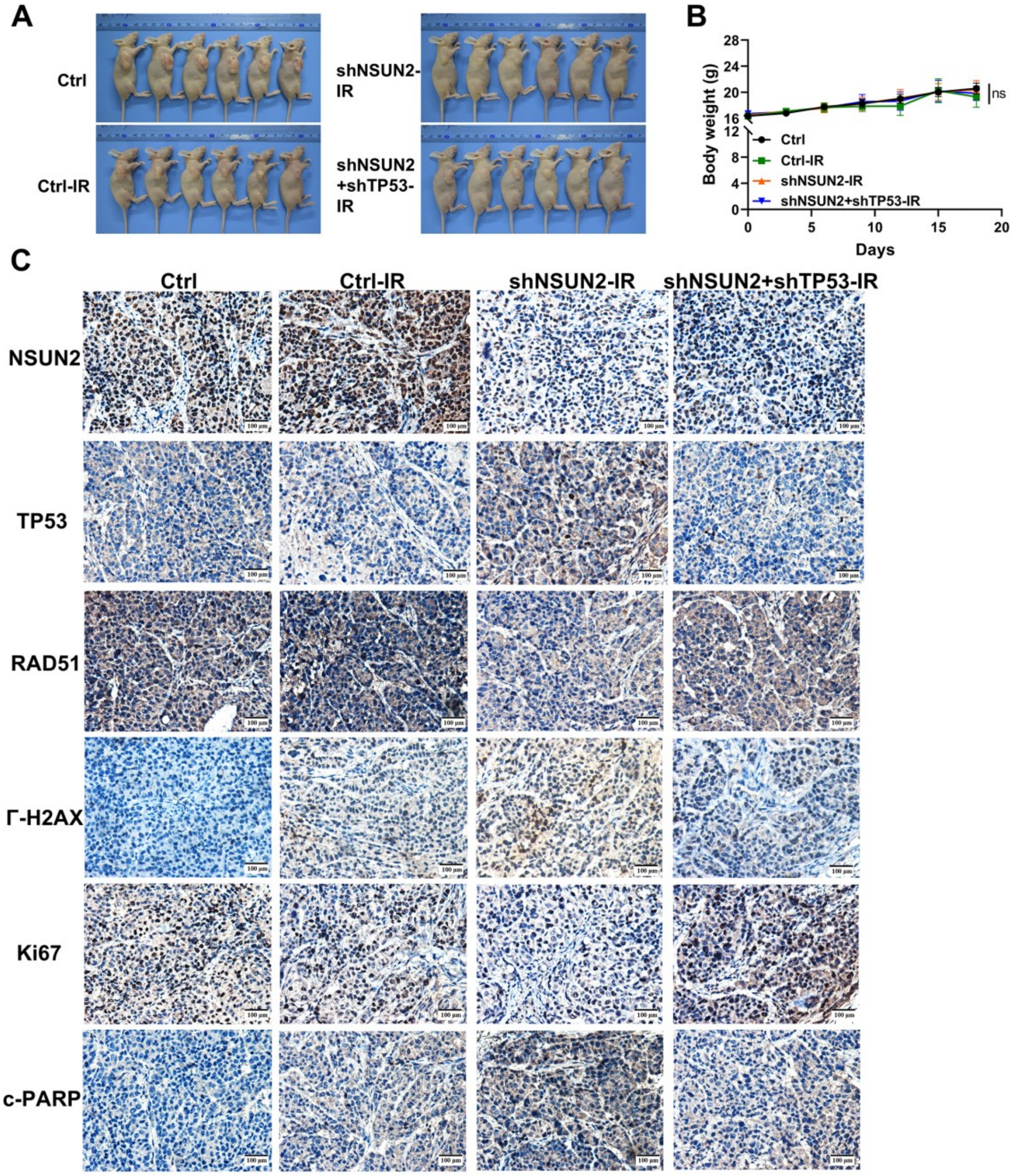

Figure S3. *In vivo* validation that NSUN2 promotes NPC radioresistance by negatively regulating TP53 expression. A. Representative images of mice. B. Body weights of mice. C. The expression of NSUN2, TP53, RAD51, γ-H2AX, Ki67 and c-PARP in tumor tissues of mice was detected by IHC (low power view).

Figure S4

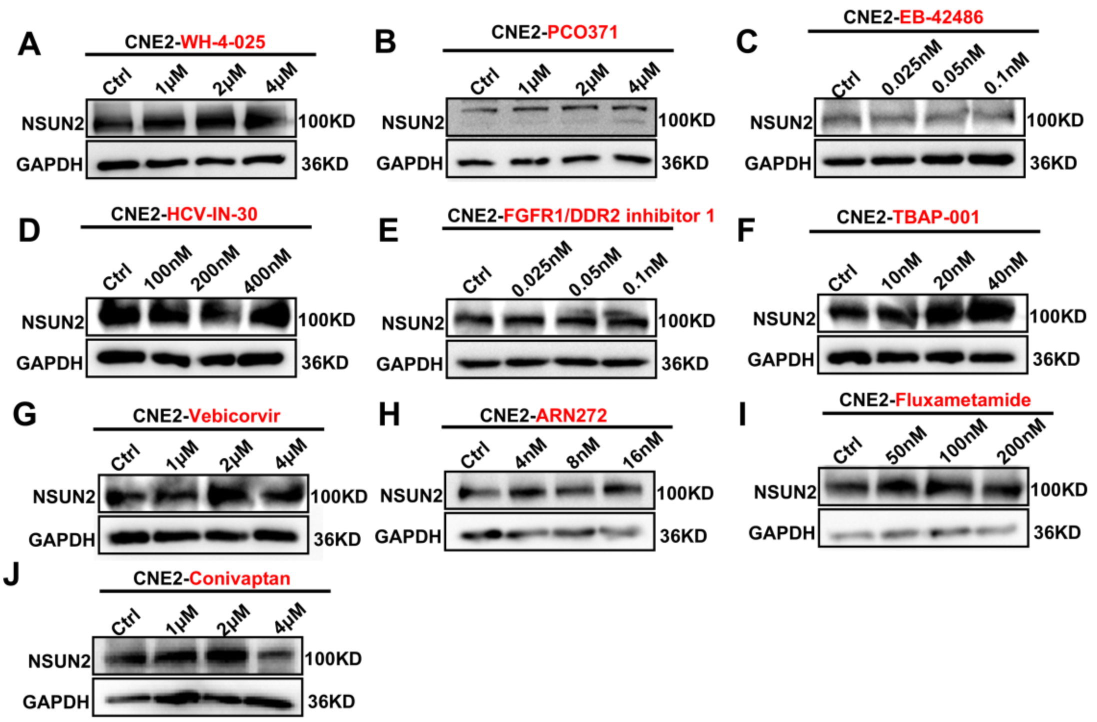

Figure S4. Screening of NSUN2 inhibitors. A. WH-4-025. B. PCO371. C. EB-42486. D. HCV-IN-30. E. FGFR1/DDR2 inhibitor 1. F. TBAP-001. G. Vebicorvir. H. ARN272. I. Fluxametamide. J. Conivaptan.

Figure S5

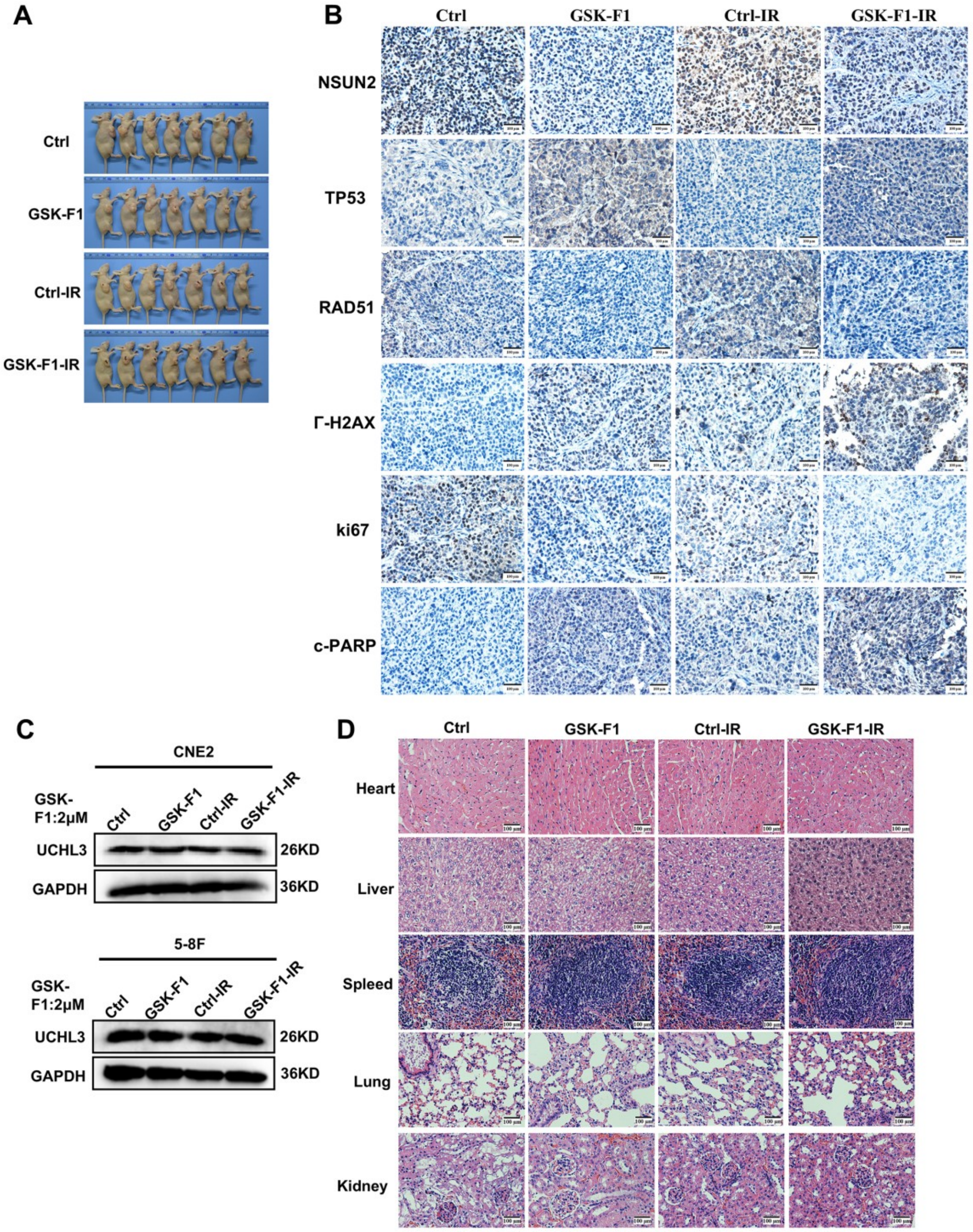

Figure S5. Effect of GSK-F1 on tumor growth, molecular expression, radiosensitization, and toxicity in mice. A. Representative images of mice treated with GSK-F1 or radiotherapy. B. The expression of NSUN2, TP53, RAD51, γ-H2AX, Ki67 and c-PARP in tumor tissues of mice was detected by IHC (low power view). C. Western blot analysis was performed to examine the effects of radiotherapy, GSK-F1,

or their combination on UCHL3 expression. D. H&E staining of major organs (heart, liver, spleen, lung, kidney) showing tissue morphology.
